# Anæsthetics

**Published:** 1905-07-29

**Authors:** 


### ANÆSTHETICS.

(*Continued from page* 295.)

FROM clinical observations on the anæsthetic effects of methyl oxide, ethyl chloride, and somnoform Hewitt[4] concludes that ethyl chloride is a fairly good substitute for nitrous oxide, and safe. Its advantages are: slightly longer anæsthesia, portability, and simplicity of administration; its disadvantages are: uncertainty of complete anæsthesia in some cases, and frequency of unpleasant after-effects, nausea, headache, and depression. The best mode of administration is by the gradual addition to a known volume of air breathed backwards and forwards into the bag. The admixture of ethyl chloride with nitrous oxide produces with extraordinary rapidity a very deep form of anæsthesia. Methyl oxide has no advantages to recommend it. Somnoform is not so good as ethyl chloride, and is not devoid of risk, two deaths having been recorded from its use.

Bampfylde Daniell[5] speaks highly of the mixture of nitrous oxide and ether for dental and short surgical operations; the patient loses consciousness with great rapidity, and an anæsthesia of from one to two and a-half minutes is procured. This mixture is largely used at the Edinburgh Dental Hospital. Dr. Daniell has for some time past been using a mixture of ethyl chloride and ether for the same purposes. It has the following advantages over the gas and ether mixtures: the patient loses consciousness more rapidly, there is little or no duskiness of the face, and the apparatus is simpler and more handy to manage. There is, however, more muscular rigidity than with gas and ether.

M. Terrier[6] gives an account of scopolamine and its use as an anæsthetic in general surgery. Its most valuable property is the production of anæsthesia for 24 to 40 hours after the return to consciousness, so that the patient does not feel any pain on the day after the operation. Three hypodermic injections are given, four, two, and one hour respectively before the operation. Occasionally a little chloroform is required at the commencement.

The clinical aspect of a scopolamised patient is that of a man in a deep natural sleep. The pulse is quickened 90 to 120, the respirations are slowed to about 15. There is slight venous congestion of the face and dilated pupils. Neither headache, nausea, nor vomiting occur, and the patient can be fed immediately after the operation. The absence of muscular relaxation is a drawback to its use in some operations. Its action is somewhat variable, chloroform being occasionally required. Dr. Terrier maintains that it is harmless, and that the deaths which have been attributed to it were due to other causes, some of the cases being acute abdominal conditions where it was considered inadvisable to give any other anæsthetic. He finds only one death recorded which might be attributable to its use, but considers this a debatable one. Professor Denucé[7] considers the sickness after chloroform largely due to the swallowed mucus and saliva impregnated with the vapour acting as an irritant in the stomach, which, according to common usage, is always kept empty before administering an anæsthetic. To combat this he has given patients copious draughts of water before operations in order to dilute the chloroform in the stomach, the results in 20 cases have exceeded his expectations, there being no sickness nor nausea after the operation.

[4] Lancet, Nov. 19-26, 1904. [5] Edin. Med. Jour., Feb. 1905. [6] Lancet, Feb. 25, 1905. [7] Ibid., Jan. 21, 1905.

